# Exploring Central Venous Thrombosis as a Rare Postinfectious Complication of Dengue: A Case Report

**DOI:** 10.1155/crnm/5368634

**Published:** 2025-04-01

**Authors:** Linda Angela Mbah, Parvinder Kaur, Dakshin Meenashi Sundaram, Shubh Mehta, Zenia Elavia, Seyi Olaniyi, Jubran Al Balushi, Jeffrin John Varghese, Archit Kumar Nigam, Mansi Singh

**Affiliations:** ^1^Department of Medicine, Veterans Affairs: Primary Care, Fort Wayne, Indiana, USA; ^2^Department of Medicine, The Crimean State Medical University Named After S. I. Georgievsky, Simferopol, Ukraine; ^3^Department of Medicine, ESIC Medical College & PGIMSR, New Delhi, India; ^4^Department of Medicine, BJ Medical College, Ahmedabad, India; ^5^Department of Medicine, Dr. D. Y. Patil Medical College, Hospital and Research Center, Sant Tukaram Nagar, Pimpri, Pune 411018, India; ^6^Department of Medicine, Obafemi Awolowo University, Ilesa, Nigeria; ^7^Department of Medicine, University College Dublin, Dublin, Ireland; ^8^Department of Medicine, Government Medical College, Thiruvananthapuram, India; ^9^Department of Radiodiagnosis, Regency Hospital, Kanpur, India; ^10^Department of Medicine, Bogomolets National Medical University, Kyiv, Ukraine

**Keywords:** anticoagulants, central venous thrombosis, dengue fever, neurological complications, thrombosis

## Abstract

Dengue fever, caused by a flavivirus transmitted through Aedes mosquitoes, presents a spectrum of clinical manifestations ranging from mild to severe. Several neurological complications, including encephalopathy and encephalitis, have been increasingly recognized. Here, we report a case of central venous thrombosis (CVT) as a postinfectious complication of dengue fever. A 38-year-old previously healthy male presented with classic dengue symptoms and later developed persistent headaches and visual disturbances. Neurological examination revealed papilledema, and imaging confirmed CVT involving the superior straight sinus. Prompt initiation of anticoagulant therapy led to gradual improvement in neurological symptoms and partial recanalization of the thrombosed sinus. Our case underscores the importance of considering thrombotic complications in dengue infections, despite the predominance of hemorrhagic manifestations. Understanding pathophysiology and appropriate management of thrombotic events in dengue fever is crucial for favorable patient outcomes.

## 1. Introduction

Dengue fever is caused by a flavivirus and is transmitted by the bite of the Aedes genus of mosquitoes in the tropics and subtropics. There are four different serotypes depending on the viral proteins on its surface, DEN-1 to DEN-4, with DEN-2 and DEN-3 being the most associated with severe disease [1]. This is especially relevant because infection by one serotype only gives immunity to that specific serotype and not any others [2]. In fact, reinfection with another serotype is associated with severe disease. Therefore, herd immunity is not attainable, and this is possibly the reason for failure of the dengue vaccine and why it affects a wide population [3]. It has a wide spectrum of clinical manifestations that range from asymptomatic infection to severe hemorrhagic disease with multisystem involvement [4]. The clinical course usually follows three phases: febrile, critical, and recovery. The febrile phase usually lasts for 3–7 days with a high fever, retro-orbital headache, prominent arthralgia, and myalgia, which is indistinguishable from other viral illnesses [5]. Clinical features suggestive of dengue include cutaneous bleeding manifestations, macular or maculopapular rash, tender hepatomegaly with elevated transaminases, and rising hematocrit with leukopenia and thrombocytopenia [6]. The critical phase is marked by the abatement of fever and heralds the onset of a systemic vascular leak that preferably involves peritoneal spaces and may last for 24–48 h. If decompensation occurs, severe shock and multiorgan failure may occur. This phase has an increased risk of bleeding and liver dysfunction [7]. The systemic vascular leak resolves in the recovery phase, and the patient experiences marked improvement in their clinical condition [6].

Today, dengue is a hyperendemic virus that puts half the world's population in jeopardy [5]. The CDC reports that dengue infects roughly 400 million people, causes symptomatic illness in 100 million, and 40,000 die every year [8]. It flourishes in tropical urban areas and is transmitted from person to person [9]. In 2009, the WHO released an updated clinical case classification scheme aimed at reducing mortality and morbidity by improving the identification of patients with advanced healthcare needs. The new classification now includes an entity known as “severe dengue fever” which includes severe organ involvement as a criterion [10]. The presence of neurological symptoms in a patient classifies them as having severe dengue fever, and a change in the spectrum of clinical manifestations with neurological manifestations has been more frequently described in recent years [11]. The incidence of encephalopathy and encephalitis, the most common neurological complications of dengue, has been estimated to be between 0.5% and 6.2%. Other neurological complications described in the literature include myelitis, acute disseminated encephalomyelitis, Guillain–Barré syndrome, myositis, hypokalemic paralysis, isolated neuropathies, and dengue-associated stroke [12].

Our case describes the rare occurrence of cerebral venous sinus thrombosis (CVST) as a complication of dengue infection. The expected complications in dengue are mostly hemorrhagic, which often results in clinicians overlooking thrombotic manifestations. This case highlights the importance of diagnosing thrombotic events in dengue infections promptly and instituting the appropriate treatment to obtain favorable outcomes.

## 2. Case Presentation

### 2.1. Patient Profile

A 38-year-old previously healthy male, presented with classic symptoms of dengue fever, including high-grade fever, intense retro-orbital headache, nausea, and severe myalgia. He had not undergone any routine check-ups or screening tests in the months leading up to the infection and reported no health concerns prior to the onset of dengue.

Physical examination revealed a blood pressure of 110/80 mmHg, fever of 102.8°F, and a pulse rate of 98 per minute. On examination, the patient was alert, oriented, and agitated and had no focal neurological deficits or neck stiffness. Laboratory investigations showed thrombocytopenia with a platelet count of 55,000/cumm, Hb–13.7 g%, and a white blood cell count of 13,500/cumm with 85% neutrophils. The coagulation panel results are normal. Liver function and renal function tests were within the normal range. Blood culture was negative for malarial parasites. The Widal test was negative. Serology ruled out infections with Hepatitis A, B, and C virus. But the patient's serology was positive for IgM antibodies against the dengue virus (Table 1).

Given lab findings and the patient's clinical presentation, a thorough workup was conducted to rule out other potential causes of thrombosis. This included negative results for common infectious conditions, such as malaria and typhoid fever, and normal coagulation parameters at the time of presentation. Although specific testing for thrombophilia factors such as Protein C and Protein S deficiencies was not performed during the acute phase, their absence was acknowledged as part of our diagnostic considerations.

A timeline of laboratory investigations showed notable changes during the patient's hospitalization. On Day 1, the platelet count was 55,000/cumm, white blood cell count was 13,500/cumm (85% neutrophils), and hemoglobin was 13.7 g%. The coagulation panel was normal, and D-dimer levels were not assessed. By Day 3, the platelet count decreased to 50,000/cumm, and the white blood cell count was 12,000/cumm. On Day 5, platelet count further declined to 40,000/cumm, white blood cell count dropped to 11,500/cumm, and D-dimer was elevated at 1200 ng/mL, indicating a possible hypercoagulable state, while the coagulation panel remained normal. By Day 7, both platelet and white blood cell counts continued to trend downward, with stable coagulation parameters.

### 2.2. Clinical Course

**Figure 1:**
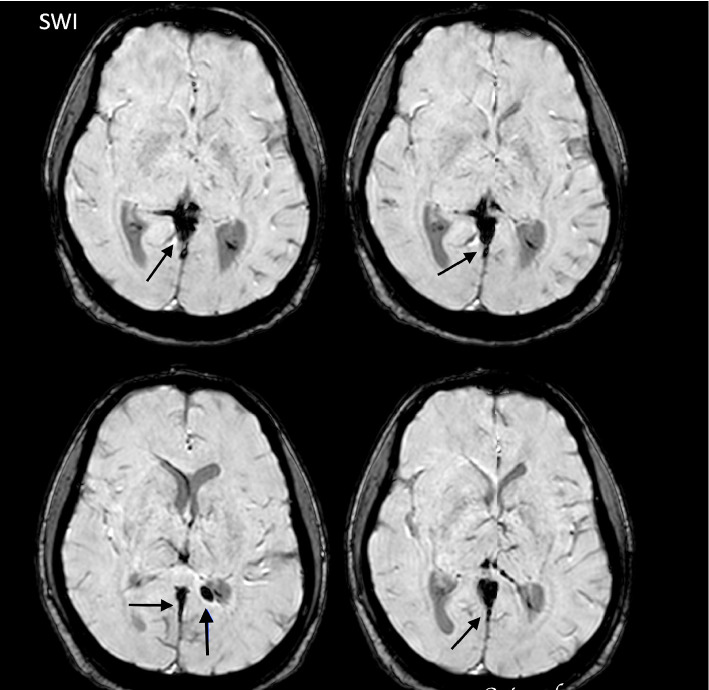
MRI brain showing thrombosis of the straight sinus.

Around the seventh day of illness, the patient developed persistent headaches, neck pain, and visual disturbances. These visual disturbances were characterized by blurring of vision and difficulty focusing. On ophthalmological examination, fundoscopy revealed papilledema, indicating elevated intracranial pressure. Despite the visual impairment, the patient's visual acuity was preserved at that time. Taking into account the clinical findings and the progression of neurological symptoms, including those evident on magnetic resonance imaging (MRI), the patient was diagnosed with CVT (Figure 1).

### 2.3. Management

Since adequate hydration is the primary treatment approach for dengue fever, the patient was effectively hydrated with crystalloid bolus according to the national guidelines for the management of dengue. The patient was promptly started on anticoagulant therapy and with subcutaneous enoxaparin 1 mg/kg/day twice daily (12 hourly). Close monitoring of platelet counts and other hematological parameters was initiated. Anticoagulation was tailored to the patient's individual risk factors and response to treatment. After Day 5 of treatment initiation, the patient's vision and headache improved.

### 2.4. Outcome

The patient showed gradual improvement in neurological symptoms over the following weeks. Repeat follow-up imaging demonstrated partial recanalization of the thrombosed sinus. He was discharged with a carefully managed anticoagulation plan and advised for follow-up.

## 3. Discussion

CVT is a thrombosis within dural venous sinus or the cerebral veins. It is relatively rare compared to venous thrombosis in other locations, such as the lower limbs. The incidence is higher in females compared to males in a 3:1 ratio, and the overall incidence is around 1% of all strokes [13]. According to the International Study on Cerebral Venous and Dural Sinuses Thrombosis (ISCVT), CVT predominantly affects younger populations, with 78% of cases occurring in patients under 50 years of age [14]. CVT is common in any condition, hereditary or acquired, that leads to a prothrombotic state. Other risk factors include infections, mechanical trauma, vasculitis, hematological disorders, systemic diseases, and certain drugs. CVT presents a wide range of signs and symptoms, thus mimicking numerous other disorders. The most common symptoms are seizures, focal neurological deficits, altered consciousness, and papilledema [15].

The prothrombotic state associated with dengue fever is a complex, multifactorial occurrence. Dehydration, which can lead to raised hematocrit, has been implicated in several cases of CVT related to dengue. Previous reports indicate that dehydration-related CVT may arise from conditions such as diabetic ketoacidosis, hyperosmolar nonketotic states, and acute gastroenteritis [16–18]. The dengue virus can actively replicate in endothelial cells, triggering the release of mediators that initiate a complex immune response. This response may result in an inversion of the CD4/CD8 ratio and cytokine overproduction, leading to immune-mediated damage to endothelial cells [19]. Additionally, the dengue virus promotes the expression of thrombomodulin in cultured endothelial cells, indicating endothelial cell activation. The infection is also associated with deficiencies in natural anticoagulants, including Protein C, Protein S, and antithrombin, further increasing the risk of thrombosis despite the concurrent thrombocytopenia that typically characterizes dengue.

In patients with dengue, the enhanced expression of thrombomodulin due to endothelial activation leads to the downregulation of activated Protein C through the formation of a complex with thrombin [18]. Low levels of Antithrombin III and Protein S are frequently observed in cases where dengue precedes CVT, potentially due to leakage of these proteins through capillary endothelium or their consumption during the immune response. Elevated levels of abnormal von Willebrand factor and soluble tissue factor have also been reported in severe dengue infections. Moreover, a shift toward a procoagulant state is indicated by increased ratios of thrombin–antithrombin to plasmin-alpha-2-antiplasmin [19]. While our patient exhibited classic dengue symptoms and thrombocytopenia, further evaluation for thrombophilia factors such as Protein C, Protein S, and Antithrombin III was not conducted. Future studies should consider these factors to better understand the thrombotic risks associated with dengue and to inform comprehensive management strategies.

Diagnosis of dengue fever is conventionally done via the detection of viral antigens, antibodies, or nucleic acids. Depending on the stage of illness, different tests are taken, for example, in febrile patients' nonstructural Protein 1 antigens are tested [20]. Up to 5 days after onset of the illness, the virus can be detected in the serum, plasma, or blood cells [21]. After this point, serology is used to confirm infection, detecting IgM antibodies if it is a first occurrence and IgG if it is a recurrence of the disease [22].

The preferred imaging modality for diagnosing CVT is MRI with MRV, with two-dimensional time-of-flight (2D TOF) MRV being the primary technique, typically revealing absent flow in the thrombosed sinus. However, the sensitivity of contrast-enhanced MRV is higher. The CVT diagnosis requires detecting thrombi in cerebral veins or sinuses, though MRV has limitations in cortical vein thrombosis and distinguishing hypoplasia from thrombosis [23]. In resource-limited settings or when contraindications exist, computed tomography (CT) with CT venography can serve as an alternative technique.

Classic signs of CVT, seen in one-third of cases, include the cord, dense triangle, and empty delta signs. CT venography confirms CVT by detecting sinus filling defects, wall enhancement, and collateral drainage. CT and CT venography provide rapid imaging with fewer artifacts, compatibility with implants, and better tolerance in claustrophobic patients. They may better visualize low-flow venous structures but have limitations, including poor skull base visualization, lower resolution for small lesions, and difficulty detecting cortical and deep vein thrombosis. Risks include contrast reactions, radiation exposure, and nephrotoxicity, limiting the use in vulnerable populations [23].

Dengue fever in general is treated with a combination of fluid resuscitation and antipyretics. Especially in the critical phase, fluid management is crucial due to the plasma leak; the recommendation suggests 50 mL/kg in 48 h of the phase [24]. Particularly in the febrile phase, studies recommend the use of paracetamol as an antipyretic. Important to note that NSAIDs should be avoided due to the thrombocytopenia and risk of bleeding associated with dengue, particularly in the case of CVT [25]. Evidence supports the use of platelet transfusion in complicated dengue, especially when there is bleeding [6]. Although there is insufficient data, immunoglobulin administration has been successful in certain studies of dengue with thrombocytopenia [26]. The treatment of choice in CVT is anticoagulation, which aims to prevent thrombus growth, facilitate recanalization, and prevent DVT or PE. Data from randomized controlled clinical trials in combination with observational data on outcomes and bleeding complications of anticoagulation support the role of anticoagulation in the treatment of CVT. LMWH is preferred in the acute treatment of CVT given its more practical administration, more predictable anticoagulation effect, lower risk of thrombocytopenia, and trends toward better outcomes in meta-analyses that do not meet the threshold for statistical significance. Thrombolytic therapy is used if clinical deterioration continues despite anticoagulation or if a patient has elevated intracranial pressure that progresses despite other approaches. The use of direct intrasinus thrombolytic techniques and mechanical therapies is only supported by case reports and small case series. If clinical deterioration occurs despite the use of anticoagulation or if the patient develops a mass effect from a venous infarction or ICH that causes intracranial hypertension resistant to standard therapies, then these interventional techniques may be considered [27].

The decision to anticoagulant dengue patients with CVST is challenging due to thrombocytopenia. Vasanthi [16] managed their patient with only parenteral fluid replacement and did not start anticoagulation therapy. Tilara et al. [17] administered anticoagulation with subcutaneous low-molecular-weight heparin in their case. Hameed et al. [18] administered therapeutic subcutaneous enoxaparin, oral rivaroxaban, and parenteral hydration. We managed the patient with parenteral fluids and subcutaneous low-molecular-weight heparin for 10 days. The prognosis is excellent, with all patients recovering completely without any sequelae. It is important to mention that although this is a rare occurrence, the prothrombotic effect of the dengue virus should be further studied as understanding its mechanism can help develop more targeted medication that eradicates the virus and reduce the risk of complications such as CVT.

## 4. Conclusion

Our case highlights the importance of recognizing CVT as a potential complication of dengue fever, particularly in patients presenting with neurological symptoms. Early diagnosis and prompt initiation of anticoagulant therapy are crucial for favorable outcomes, emphasizing the need for heightened awareness and comprehensive management strategies in dengue-infected individuals prone to thrombotic events.

## Figures and Tables

**Figure 2:**
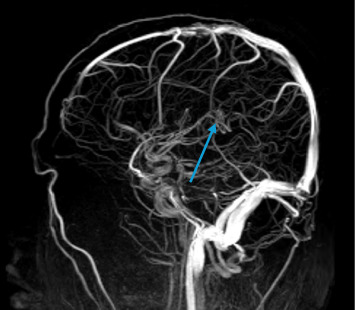
MR venogram showing isolated straight sinus thrombosis.

**Table 1:** Laboratory investigations of the patient on admission.

Platelet count	55,000/cumm
Hemoglobin	13.7 g%
WBC	13,500/cumm
Neutrophil percentage of WBC	85%
Coagulation panel	Normal
Liver function	Normal
Renal function	Normal
Blood culture for malarial parasites	Negative
Widal test	Negative
Hepatitis A	Negative
Hepatitis B	Negative
Hepatitis C	Negative
Serology for anti-dengue IgM antibodies	Positive

## Data Availability

The data are available on request from the corresponding author.
